# Time Series Forecasting of Motor Bearing Vibration Based on Informer

**DOI:** 10.3390/s22155858

**Published:** 2022-08-05

**Authors:** Zhengqiang Yang, Linyue Liu, Ning Li, Junwei Tian

**Affiliations:** 1 School of Computer Science and Engineering, Xi’an Technological University, Xi’an 710021, China; 2 School of Electrical Engineering, Xi’an University of Technology, Xi’an 710048, China; 3 School of Mechatronic Engineering, Xi’an Technological University, Xi’an 710021, China

**Keywords:** motor bearing vibration, time series forecasting, Informer, Transformer, random search

## Abstract

Electric energy, as an economical and clean energy, plays a significant role in the development of science and technology and the economy. The motor is the core equipment of the power station; therefore, monitoring the motor vibration and predicting time series of the bearing vibration can effectively avoid hazards such as bearing heating and reduce energy consumption. Time series forecasting methods of motor bearing vibration based on sliding window forecasting, such as CNN, LSTM, etc., have the problem of error accumulation, and the longer the time-series forecasting, the larger the error. In order to solve the problem of error accumulation caused by the conventional methods of time series forecasting of motor bearing vibration, this paper innovatively introduces Informer into time series forecasting of motor bearing vibration. Based on Transformer, Informer introduces ProbSparse self-attention and self-attention distilling, and applies random search to optimize the model parameters to reduce the error accumulation in forecasting, achieve the optimization of time and space complexity and improve the model forecasting. Comparing the forecasting results of Informer and those of other forecasting models in three publicly available datasets, it is verified that Informer has excellent performance in time series forecasting of motor bearing vibration and the forecasting results reach $10^{-2}$∼$10^{-6}$.

## 1. Introduction

Electric energy plays an essential role in human life and technological development. The motor is the core equipment of the power station; therefore, monitoring the motor conditions can effectively avoid the occurrence of hazards and improve the safety. In recent years, there have been many bearing health monitoring technologies, such as noise monitoring, temperature monitoring, current detection and vibration monitoring, etc. [1,2,3,4,5]. Among them, vibration monitoring can detect, locate and distinguish faults before serious failures of bearings occur. For the research of bearing fault diagnosis and bearing remaining useful life (RUL) prediction, time series forecasting of motor bearing vibration is a crucial prerequisite step. Therefore, it is of great significance to study the vibration prediction of motor bearings. The vibration signal of the motor bearing obtained by the sensor can reflect the fault characteristics [6,7,8]. Different fault types will produce different frequencies, amplitudes and corresponding vibrations in different parts of the apparatus [9]. The fault prediction based on motor bearing vibration data, which is applied to the monitoring of the sensing technology, can effectively avoid hazards such as bearing heating, thus saving maintenance costs [10].

Time series forecasting of motor bearing vibration is to determine the possibility of future failure by analyzing the historical data of its components. Conventional methods can be broadly classified into three main categories: classical time series forecasting and its optimization methods, forecasting methods based on sliding window and forecasting methods based on encoder–decoder structure.

Classical time series forecasting methods [11,12] achieve forecasting mainly through fixed time dependence and the single factor. The time series analysis method proposed by Box et al. [13] predicted the subsequence data series based on the known data series. Nikovski et al. [14] verified by experiments that classical time series forecasting methods have some advantages in the single factor short-term forecasting. Classical time series forecasting methods rely on linear relationships and do not include complex nonlinear dynamic models. This property makes the learning ability and expression ability of such methods inadequate and the forecasting results are poor in the face of complex and weak periodic motor bearing vibration data.

Time series forecasting methods of motor bearing vibration based on sliding window forecasting, such as CNN [15], RNN [16], LSTM [17] and other algorithms, were able to forecast nonlinear functions and dynamic dependency [18,19], which brought new results for complex time series forecasting containing multiple covariate inputs. Time series forecasting based on CNN and their improved models have been widely used. Shao et al. [20] used a light-weight 1D-CNN model combined with an auto-encoder structure and adopted a correlation alignment (CORAL) method to reduce domain offset. Luo et al. [21] used the conditional mutual information method to filter variables and the Pair-Copula model by incorporating the kernel density estimation method to address the limitation that the traditional Copula model can only handle two-dimensional variables and finally chose to combine with SVM and BP neural network to realize the data prediction. Carroll et al. [22] used artificial neural networks, SVM and logistic regression methods to demonstrate that the prediction of gearbox failures can be achieved using vibration data training models. Rahmoune et al. [23] applied the residual neural network model to a gas turbine system to predict the vibration frequency of the bearing through the vibration frequency data obtained by the sensor at the bearing. As a model specializing in forecasting series applied to time series forecasting, RNN has its advantages. Senjyu et al. [24] used RNNs, obtaining the input and output data of the network by differential calculations, to better predict the power variation of wind turbine bearings. Liu et al. [25] used RNN in the form of auto-encoders to diagnose bearing faults and forecast the rolling bearing data from the previous cycle to the next cycle through a GRU-based nonlinear predictive denoising auto-encoder (GRU-NP-DAE). Che et al. [26] proposed a fault prediction model based on the RNN variant model, Gate recurrent unit (GRU) and hybrid auto-encoder fault prediction model, which introduced the original signals into a multi-layer gate recurrent unit model to achieve time series forecasting and then achieved fault detection by the variational auto-encoders and stacked denoising auto-encoders. The effectiveness of this method was verified by the bearing dataset of Case Western Reserve University. The LSTM model solved the long-term dependence problem of general RNN models and further improved the time series forecasting. Ma et al. [27] proposed a model based on optimizing maximum correlation kurtosis deconvolution (MCKD) and LSTM network for time series forecasting of motor bearing vibration to realize early bearing fault warnings. Liu et al. [28] proposed a multilayer long short-term memory-isolation forest model (MLSTM-iForest) to predict the bearing temperature in the future and then input the calculated deviation index of the predicted bearing temperature into iForest to realize bearing fault early warning. ElSaid et al. [29] proposed to improve the LSTM cell structure using the ant colony optimization algorithm (ACO) for forecasting engine data and the new model presented an improvement of 1.35%. Fu et al. [30] used CNN to extract features and then used LSTM for gearbox bearing forecasting to achieve bearing high speed-side monitoring and super high temperature warning. Based on the sliding window forecasting methods, there was an error accumulation problem in time series forecasting. If these models were then used in combination with other methods, the training time would become longer, so timely forecasting of motor bearing vibration could not be achieved. Some of the above methods are suitable for small datasets and the forecasting results are not satisfactory for big data.

Time series forecasting methods of motor bearing vibration based on encoder–decoder structure, such as the Transformer model [31], used the attention mechanism to improve model training speed, which was suitable for parallelized calculation and higher than RNN in accuracy and performance. The unique output mechanism of the Transformer model can largely reduce the error accumulation during forecasting. Tang et al. [32] used discrete wavelet transform (DWT) and continuous wavelet transform (CWT) to convert vibration signals into a time-frequency representation (TFR) map and performed preliminary prediction analysis of TFR map by multiple individual ViT models [33] which had better results compared with integrated CNN and individual ViT. Zhang et al. [34] proposed a self-attention-based perception and prediction framework based on Transformer, called DeepHealth. Xu et al. [35] proposed a prediction model (HNCPM) that combines encoder, GRU regression module and decoder, through which the prediction of vibration data is realized. This model deploys an enhanced attention mechanism to capture global dependency from vibrational signals to forecast future signals and predict facility health. However, the training time of time series forecasting methods of motor bearing vibration based on encoder–decoder structure was long; what is more, these above research methods used a single dataset, which could not well illustrate the robustness of the proposed methods.

Based on the above problems and analysis, in this paper, the Informer model [36] is innovatively introduced into the prediction of motor bearing vibration and a time series forecasting method of motor bearing vibration based on random search [37] to optimize the Informer model is proposed. In this paper, we mainly focus on solving the problems of error accumulation, time and space complexity, optimization of model parameters and singleness of the dataset. Three publicly available datasets are selected and divided to form ten new datasets to compare the robustness of different models. The structure of Informer is improved for time series forecasting of motor bearing vibration and the parameters of Informer are optimized by random search. The main contributions of this paper are summarized as follows: (1) Informer is innovatively introduced into time series forecasting of motor bearing vibration. (2) For time series forecasting of motor bearing vibration, Informer is optimized and random search is used to optimize the model parameters to improve the model prediction effect.

The rest of this paper is organized as follows. Section 2 describes CNN, Deep RNNs, LSTM and Transformer and illustrates the problems of applying the above four models to time series forecasting of motor bearing vibration. Section 3 introduces Informer and its model optimization. Section 4 presents three publicly available datasets, compares the forecasting results of Informer with the other four models, illustrates the experimental results and conducts analyses. Section 5 presents the conclusion.

## 2. Conventional Methods Applied to Time Series Forecasting of Motor Bearing Vibration

This section introduces four models (CNN, Deep RNNs, LSTM and Transformer) applied to time series forecasting of motor bearing vibration and analyzes their limitations.

### 2.1. Convolutional Neural Networks (CNN)

The nonlinear mapping through the activation function solves the problems that classical time series prediction methods cannot incorporate exogenous variables and they rely on linear relationships. The motor bearing vibration data contains positive and negative values and the values fluctuate around 0. According to the characteristics of this motor bearing vibration data, this paper selects the tanh function as the activation function of CNN, which maps the input values to the range (−1,1). The equation is as follows:

(1) $$tanh\left(x\right)=\frac{e^{x}-e^{-x}}{e^{x}+e^{-x}}$$

There are some common activation functions: The softmax function is as follows:

(2) $$softmax\left(x_{i}\right)=\frac{e^{x_{i}}}{\sum_{i=1}^{C}e^{x_{i}}}$$

where *C* is the length of the input sequence and $x_{i}\;\left(0\leq i\leq C\right)$ is the *i*-th element in the input sequence.

The ELU function is as follows:

(3) $$ELU\left(x\right)=\left\{\begin{array}{c} x,x>0\\ a(e^{x}-1),x\leq 0 \end{array}\right.$$

where *a* is a positive decimal close to 0.

### 2.2. Deep Recurrent Neural Networks (Deep RNNs)

Deep RNNs [38,39] as a model specially dealing with series, in view of the long sequence and big data characteristics of motor bearing vibration data, this paper selects an input window of 100 to verify the long sequence forecasting effect of this model. According to the motor bearing vibration data characteristics described in Section 2.1, the tanh function (Equation (1)) is selected as the activation function of Deep RNNs. The input data of the cell at the *i*-th layer and *t*-th time come from two directions, one is the output $h_{t}^{i-1}$ from the (i−1)-th layer and its equation is as follows:

(4) $$h_{t}^{i-1}=f\left(W^{i-1}h_{t-1}^{i-1}+U^{i-1}h_{t}^{l-2}\right)$$

The other comes from the *i*-th layer and (t−1)-th time memory data and its equation is as follows:

(5) $$h_{t-1}^{i}=f\left(W^{i}h_{t-2}^{i}+U^{i}h_{t-1}^{l-1}\right)$$

The equation of the output $h_{t}^{i}$ of the cell is as follows:

(6) $$h_{t}^{i}=f\left(W^{i}h_{t-1}^{i}+U^{i}h_{t}^{l-1}\right)$$

### 2.3. Long Short-Term Memory (LSTM)

Generally, the frequency of collecting motor bearing vibration data is relatively large and some values of the adjacent data collected in a very short period of time are very small, resulting in data redundancy in the process of learning. LSTM selects and discards part of the information through the forget gate and determines how much historical information enters, i.e., filters extremely similar adjacent motor bearing vibration data while preserving the trend of the original motor bearing vibration data. The forget gate will read $h_{t-1}$ and $x_{i}$ and output a value between 0 and 1 to each number in the cell state $C_{i-1}$. The equation is as follows:

(7) $$f_{t}=\sigma \left(W_{f}\times \left[h_{t-1},x_{t}\right]+b_{f}\right)$$

where $h_{t-1}$ is the output of the previous cell; $x_{t}$ is the input of the current cell; σ is the tanh function (Equation (1)). Update the old cell state with the following equation:

(8) $$i_{t}=\sigma \left(W_{i}\times \left[h_{t-1},x_{t}\right]+b_{i}\right)$$

(9) $$\tilde{C_{t}}=tanh\left(W_{C}\times \left[h_{t-1},x_{t}\right]+b_{C}\right)$$

The result is output through the output gate and the equation is as follows:

(10) $$o_{t}=\sigma \left(W_{o}\times \left[h_{t-1},x_{t}\right]+b_{o}\right)$$

(11) $$h_{t}=o_{t}\times tanh\left(C_{t}\right)$$

### 2.4. Transformer

Motor bearings are extremely delicate components in machines; for various reasons, only a small fraction of them can reach their design life [40,41]. Therefore, it is important to perform long-term vibration detection of motor bearings as well as to record recent abnormal vibrations. Transformer model based on Multi-head self-attention has the ability to simultaneously model long-term and short-term time series features, which is applicable to long-term motor bearing vibration data while learning short-term vibration features. This paper selects an input window of 100 to verify the Transformer’s ability to model time series data. The equation of the multi-head self-attention mechanism is as follows:

(12) $$MultiHead\left(Q,K,V\right)=Concat\left(head_{1},...,head_{n}\right)W^{o}$$

where $head_{i}=Attention\left(QW_{i}^{Q},KW_{i}^{K},VW_{i}^{V}\right),W_{i}^{Q},W_{i}^{K}$ and $W_{i}^{V}$ are the parameters that can be learned. The attention method is as follows:

(13) $$Attention\left(Q,k,V\right)=softmax\left(\frac{QK^{T}}{\sqrt{d_{k}}}\right)V$$

where the softmax function is shown in Equation (2). *K* is the key matrix, *Q* is the query matrix and *V* is the value matrix. The equation of layer normalization is as follows:

(14) LayerNorm=(x+SubLayer(x))

In order to ensure that the decoder cannot see those inputs after the current moment, Transformer uses an attention mechanism with a mask to ensure consistent behavior during training and forecasting. To solve the problem that the relative position of the input is disrupted, Transformer adds the position encoding of the input information to the input information at the Positional Encoding layer before sending the input into the self-attention layer. The specific calculation equation is as follows:

(15) $$PE\left(pos,2i\right)=sin\left(\frac{pos}{2L^{\frac{2i}{d}}}\right)$$

(16) $$PE\left(pos,2i+1\right)=cos\left(\frac{pos}{2L^{\frac{2i}{d}}}\right)$$

where pos is the position of the current word in the whole input sequence. *i* is the dimension of the current calculated value (maximum is *d*). *d* is the dimension of the input sequence. *L* is the length of the sequence.

### 2.5. Insufficiency of Conventional Methods Applied to Time Series Forecasting of Motor Bearing Vibration

#### 2.5.1. Insufficiency of Sliding Window Forecasting

**Figure 1:**
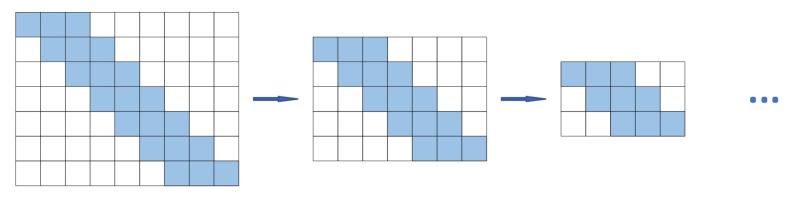
Sliding window mechanism.

**Figure 2:**
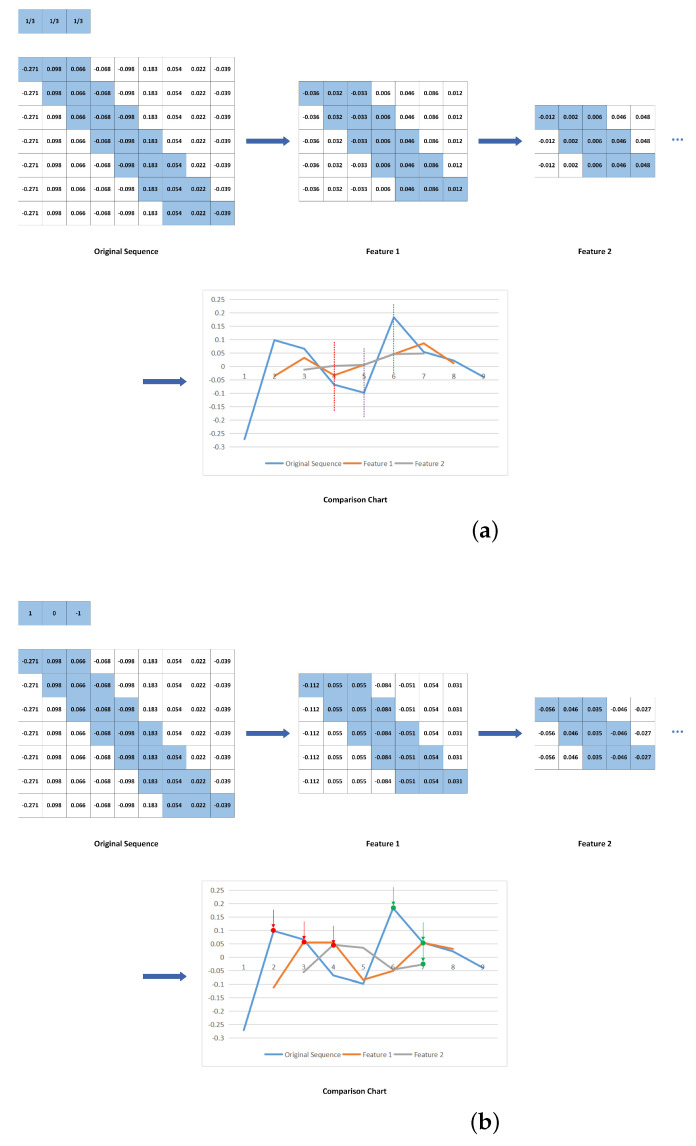
Deviations caused by sliding window: (**a**) Deviations lead to ambiguity of feature series; (**b**) Deviations lead to offset of feature series.

There are some defects in the forecasting method of motor bearing vibration time series based on sliding window mechanism model [42]. The commonly used sliding window leads to spatial and temporal deviations in the feature map or the feature sequence. This deviation leads to ambiguity and offset in the feature sequence. The commonly used sliding window is applied to the motor vibration data with long sequence and big data characteristics, which will cause the error to accumulate continuously, the sliding window mechanism, as shown in Figure 1. Real bearing vibration data [43] is chosen for illustration, as shown in Figure 2.

Meanwhile, time series forecasting methods based on CNN, Deep RNNs and LSTM of motor bearing vibration also have their own insufficiency. The time series forecasting method based on CNN captures short-term local dependency; thus, its forecasting effect depends on the degree of correlation of the short-term data. Normal bearing vibrations have a certain periodicity in the short term, but this model could not forecast abnormal vibrations without regularity. Although the Deep RNNs can enhance its expression ability, this model is calculationally intensive and the training process is time-consuming and is unable to give timely forecasting results when facing new data, i.e., it cannot give ideal forecasting results for future abnormal vibrations. In addition, as the scale and depth of the Deep RNN model increase, learning will become more difficult. Therefore, when faced with motor bearing vibration data with big data characteristics, building a matching Deep RNN is still a problem that needs to be solved. LSTM also has the problem of calculational time consumption and the disadvantage of parallel processing. LSTM is not able to give reasonable prediction results because of the poor correlation between the abnormal vibration data and the previous data.

#### 2.5.2. Insufficiency of Transformer

Position encoding is an important part of Transformer, which is divided into absolute position encoding and relative position encoding. Currently, relative position encoding operates on the attention matrix before softmax, which has a theoretical drawback [44,45]. The attention matrix with relative location information is a probability matrix with each row summed equal to 1. For Transformer, self-attention implements the interaction between tokens and the same input indicates that each $v_{t-1}$ is the same. According to the description in Section 2.3, some values of the motor bearing vibration data collected in a very short period of time differed very little. That is, the output results for each location of the model are always the same or extremely similar data due to the accuracy problem resulting in the same output results.

(17) $$o_{i}=\sum_{j}a_{i,j}v_{j}=\sum_{j}a_{i,j}v=\left(\sum_{j}a_{i,j}\right)v=v$$

where $o_{i}$ is the output value; $a_{i,j}$ is the softmax value (shown in Equation (2)); $\sum_{j}a_{i,j}=1$ causes the sum of each row of the attention matrix to be 1; $v_{j}$ is the value.

Transformer also has the defects of large amount of calculation and long training time. Compared with CNN and RNN, Transformer has a weaker ability to acquire local information.

## 3. Informer Applied to Time Series Forecasting of Motor Bearing Vibration

**Figure 3:**
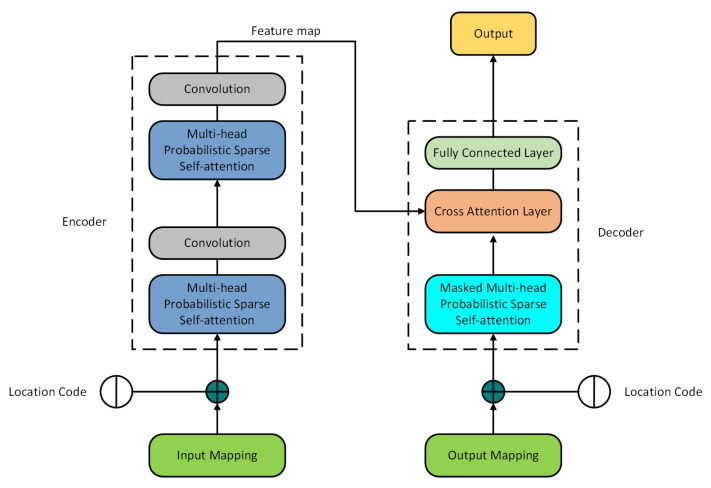
Informer structure [36].

This section introduces Informer applied to time series forecasting of motor bearing vibration, describes the insufficiency of using Informer directly and optimizes Informer. Informer structure, as shown in Figure 3.

### 3.1. Informer Introduction

Informer adds positional encoding to the data input to ensure that the model can capture the correct order of the input sequence. The location encoding is divided into Local Time Stamp and Global Time Stamp. The equation of the Local Time Stamp is shown in Equations (15) and (16).

After the encoding steps, the input data into the encoder layer can be obtained, as shown below:

(18) $$x_{i}^{t}=\alpha u_{i}^{t}+PE_{\left(L\times (t-1)+i\right)}+\sum_{P}{\left[SE_{\left(L\times (t-1)+i\right)}\right]}_{P}$$

where $u_{i}$ is the original data sequence, i∈[1,2,...,L]; *L* is the length of the data sequence; *t* is the number of series; α is a factor to balance the size between the mapping vector and the position encoding and is taken as 1 in the case that the input sequence has been standardized.

Informer introduces ProbSparse self-attention, which first calculates the KL divergence of the *i*-th query and the uniformly distributed query to obtain the difference degree and then calculates the sparsity score. The formula for calculating KL divergence is as follows:

(19) $$KL\left(q||p\right)=\sum_{j=1}^{L_{K}}\frac{1}{L_{K}}\ln\frac{\frac{1}{L_{K}}}{\frac{k(q_{i},k_{j})}{\sum_{l}k(q_{i},k_{l})}}=\log\sum_{l=1}^{L_{K}}e^{\frac{q_{i}k_{l}^{T}}{\sqrt{d}}}-\frac{1}{L_{K}}\sum_{j=1}^{L_{K}}\frac{q_{i}k_{j}^{T}}{\sqrt{d}}-logL_{K}$$

where p($k_{j}$|$q_{i}$) is the probability distribution of the attention query for all keys; q($k_{j}$|$q_{i}$)=$\frac{1}{L_{K}}$ is the uniform distribution; *d* is the dimension of the input sequence after mapping; $L_{K}$ is the sequence length; *k*($q_{i}$, $k_{j}$) is the intermediate value of the *i*-th query and the *j*-th key when performing the softmax (Equation (2)) calculation. The sparsity score metric of the *i*-th query is as follows:

(20) $$M\left(q_{i},K\right)=\log\sum_{l=1}^{L_{K}}e^{\frac{q_{i}k_{l}^{T}}{\sqrt{d}}}-\frac{1}{L_{K}}\sum_{j=1}^{L_{K}}\frac{q_{i}k_{j}^{T}}{\sqrt{d}}$$

Based on the above metrics, each key focuses on only *u* dominant queries, namely ProbSparse self-attention:

(21) $$Attention\left(Q,K,V\right)=softmax\left(\frac{\bar{Q}K^{T}}{\sqrt{d}}\right)V$$

where $\bar{Q}$ is a sparse matrix with the same shape as *Q*, which contains only the first *u* queries under the sparsity measure $M(q_{i},K)$, which has the following properties of the upper and lower bounds:

(22) $$\log L_{K}<M\left(q_{i},K\right)<\max_{j}\left\{\frac{q_{i}k_{j}^{T}}{\sqrt{d}}\right\}-\frac{1}{L_{K}}\sum_{j=1}^{L_{K}}\frac{q_{i}k_{j}^{T}}{\sqrt{d}}+\log L_{K}$$

where $\max_{j}\left\{\frac{q_{i}k_{j}^{T}}{\sqrt{d}}\right\}$ replaces $\log\sum_{l=1}^{L_{K}}e^{\frac{q_{i}k_{l}^{T}}{\sqrt{d}}}$ in the original equation to obtain the approximation result of *M*, shown as follows:

(23) $$\bar{M}\left(q_{i},K\right)=\max_{j}\left\{\frac{q_{i}k_{j}^{T}}{\sqrt{d}}\right\}-\frac{1}{L_{K}}\sum_{j=1}^{L_{K}}\frac{q_{i}k_{j}^{T}}{\sqrt{d}}$$

**Figure 4:**
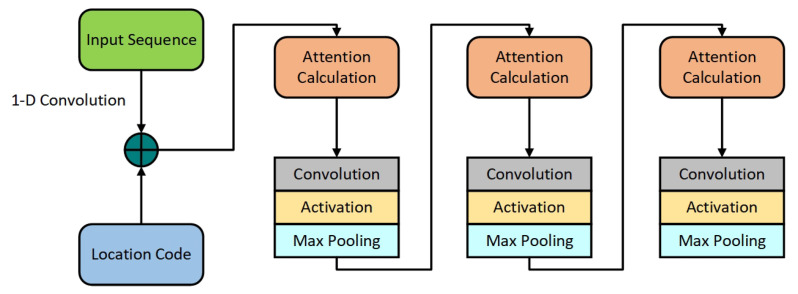
Self-attention distilling [36].

Informer introduces the self-attention distilling, as shown in Figure 4, which adds convolution, activation and maximum pooling operations between each encoder and decoder layer to reduce the length of the input sequence of the previous layer by half, thus solving the problem of occupying too much memory when the input sequence is long. The equation is as follows:

(24) $$X_{j+1}^{t}=MaxPool\left(ELU\left(Conv1d\left({\left[X_{j}^{t}\right]}_{AB}\right)\right)\right)$$

where $X_{j+1}^{t}$ is the output of the multi-headed ProbSparse self-attention layer in this layer; ${\left[X_{j}^{t}\right]}_{AB}$ is the calculation result of the multi-headed ProbSparse self-attention layer in the previous layer; ELU (Equation (3)) is used as the activation function.

Informer model uses batch generation forecasting to directly output multi-step forecasting results, thus improving the speed of long series forecasting. The equation is as follows:

(25) $$X_{feed_{decoder}^{t}}=Condat\left(X_{token}^{t},X_{0}^{t}\right)\in R^{\left(L_{token}+L_{y}\right)\times d_{model}}$$

where $X_{0}^{t}$ is the placeholder (predicted value); $X_{token}^{t}\in R^{L_{token}\times d_{mode}}$ is the start token; $L_{token}$ is the length of the sequence of start tokens; $L_{y}$ is the length of the predicted sequence; $d_{model}$ is the model dimension.

### 3.2. Informer Optimization

**Figure 5:**
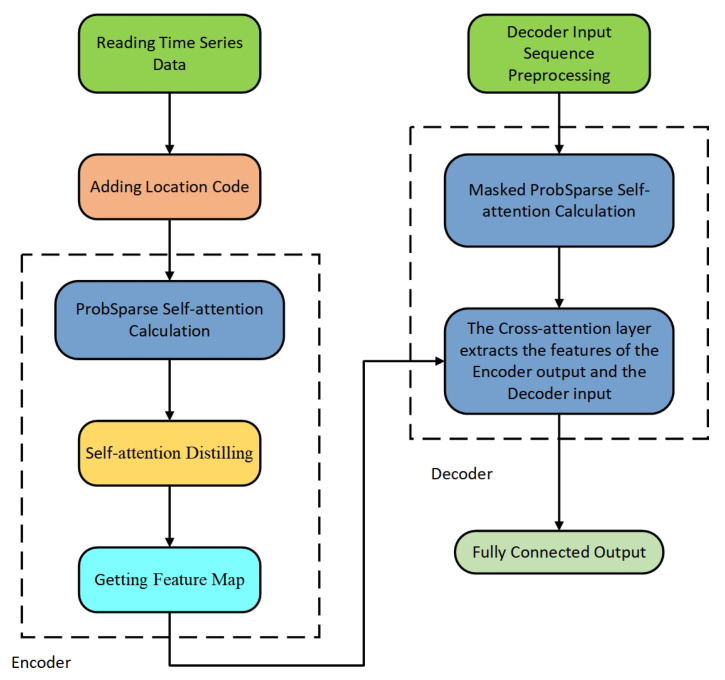
Time series forecasting methods of motor bearing vibration based on Informer.

Informer forms sparse attention through query and key in ProbSparse self-attention to reduce the computational complexity of motor vibration feature learning. In Equation (23), $L_{Q}=L_{K}=L$, so that the total time complexity and space complexity are O(LlnL). In self-attention distilling, the input of the cascade layer is halved to deal with the super-long input sequence and alleviate the accumulative error problem of the classical neural network model. Zhou et al. [36] predicted results of long-series based on ETT, ECL and ELU activation function to be $10^{-1}$, which did not meet the requirements of time series forecasting of motor bearing vibration results. This paper optimizes the Informer model based on the vibration data of motor bearings. Time series forecasting methods of motor bearing vibration based on Informer, as shown in Figure 5.

**Figure 6:**
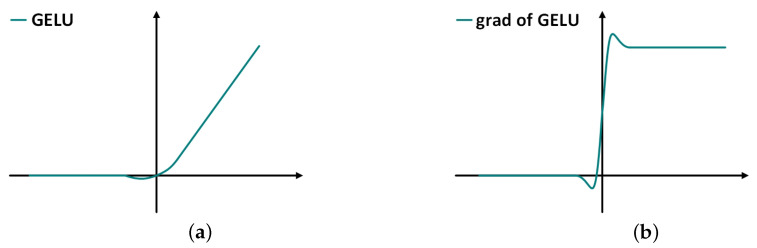
(**a**) GELU activation function image; (**b**) The corresponding derivative image of GELU activation function.

The motor bearing vibration data contain positive and negative values and the values fluctuate around 0. According to the GELU activation function image and its corresponding derivative image, it can be seen that, compared with the ELU activation function, the GELU activation function is more consistent with the motor bearing vibration data characteristics. Therefore, GELU is chosen as the activation function of Informer in this paper. The GELU activation function image and its corresponding derivative image is shown in Figure 6. The equation of the GELU activation function is as follows:

(26) $$GELU\left(x\right)=xP\left(X\leq x\right)=x\Phi \left(x\right)\approx 0.5x(1+tanh\left[\sqrt{\frac{2}{\pi }}\left(x+0.044715x^{3}\right)\right])$$

The three datasets used in this paper have high sampling frequency. For this feature, the time feature code was selected as hour, which can realize the training and prediction of the model for long-sequence data. The verification prediction length has 500 sample points and the results showed that the model was able to process and forecast the data series with long series and big data characteristics. After several tests, Informer converged at epoch 10 for all three datasets. According to the characteristics of motor bearing vibration data, the conventional method cannot complete the model training quickly when facing the newly generated data. Therefore, under the premise of ensuring the accuracy of prediction, this paper reduces the model size and the model calculation running time and selects two encoder layers and one decoder layer.

In this paper, the hyper parameter λ of Informer was optimized for time series forecasting of motor bearing vibration data. Usually the ultimate goal of the learning algorithm is to find a function that satisfies the minimum loss function and the so-called learning of the algorithm is the learning of the hyper parameter. In this paper, random search was used to optimize the hyper parameter λ to determine a better model [34,46,47,48]. The hyper parameter λ is as follows:

(27) $$\lambda \left(*\right)=argminE_{x∼G_{x}}\left[L\left(x;A_{\lambda }\left(X^{train}\right)\right)\right],\lambda \in \Lambda$$

(28) $$\lambda \left(*\right)\approx argminmeanL(x;A_{\lambda }\left(X^{train}\right)),\lambda \in \Lambda ,x\in X^{valid}$$

(29) ≡argminΨ(λ),λ∈Λ

(30) $$\approx argmin\Psi \left(\lambda \right)\equiv \hat{\lambda },\lambda \in \left\{\lambda^{\left(1\right)}...\lambda^{\left(S\right)}\right\}$$

where Ψ is the hyper parametric response function. $\left\{\lambda^{\left(1\right)}...\lambda^{\left(S\right)}\right\}$ is the experimental set.

(31) $$\Psi^{valid}\left(\lambda \right)=mean_{x\in X^{valid}}L\left(x;A_{\lambda }\left(X^{train}\right)\right)$$

(32) $$\Psi^{test}\left(\lambda \right)=mean_{x\in X^{test}}L\left(x;A_{\lambda }\left(X^{train}\right)\right)$$

where $\Psi^{valid}$ denotes the performance of the validation set; $\Psi^{test}$ denotes the performance of the testing set.

The equation of the estimated variance of the mean is as follows:

(33) $$V^{valid}\left(\lambda \right)=\frac{\Psi^{valid}\left(\lambda \right)\left(1-\Psi^{valid}\left(\lambda \right)\right)}{|X^{valid}|-1}$$

(34) $$V^{test}\left(\lambda \right)=\frac{\Psi^{test}\left(\lambda \right)\left(1-\Psi^{test}\left(\lambda \right)\right)}{|X^{test}|-1}$$

When multiple parameter values are close to optimal and do not differ significantly, they are determined by weighting the best probability in their particular $\lambda^{\left(S\right)}$. In [34], it was proposed that $X^{valid}$ is a finite sample of $G_{x}$; thus, the testing set score of the best model in $\lambda^{\left(1\right)}...\lambda^{\left(S\right)}$ is a random number *Z* which is modeled by a Gaussian mixture model with $\mu_{S}=\Psi^{test}\left(\lambda^{S}\right)$ (the mean of *S*) and $\sigma_{S}^{2}=V^{test}\left(\lambda^{S}\right)$ (the variance of *S*). The weights are:

(35) $$w_{S}=P\left(Z^{S}<Z^{S^{{}^{\prime }}},\forall S^{{}^{\prime }}\neq S\right),Z^{i}∼N\left(\Psi^{valid}\left(\lambda^{i}\right),V^{valid}\left(\lambda^{i}\right)\right)$$

The mean and standard error of *Z* in the optimal model are:

(36) $$\mu_{Z}=\sum_{S=1}^{S}w_{S}\mu_{S}$$

(37) $$\sigma_{Z}^{2}=\sum_{S=1}^{S}w_{S}\left(\mu_{S}+\sigma_{S}^{2}\right)-\mu_{Z}^{2}$$

By the above method, the hypothesis validation score $Z^{S}$ is continuously extracted from the normal distribution, its testing score is calculated, the optimal estimate value is selected and the optimal parameters are determined. In the face of time series forecasting of motor bearing vibration, the best forecasting result is obtained when the batch size is 16 and the learning rate is 0.0001 in Informer. When the learning rate is too large, the model will oscillate near the optimal solution, and when it is too small, the model will converge too slowly. The choice of dropout is related to whether the model excessively considers the data correlation and noise data. In order to prevent the model from being over-fitted which leads to the reduction of the model robustness, the best result is obtained when dropout is selected as 0.02 after the test. The parameters of Informer used in this paper are shown in Table 1.

## 4. Experiments and Results

### 4.1. Dataset Introduction

#### 4.1.1. Case Western Reserve University Bearing Dataset

This paper uses a publicly available bearing dataset from the Bearing Data Center at Case Western Reserve University (CWRU) in the United States [49]. The experimental rig used to acquire this dataset consisted of a 2 hp motor, a torque transducer/encoder, a dynamometer and control electronics. An accelerometer was placed above the bearing seat of the motor drive side and the fan side and a 16-channel DAT recorder was used to collect vibration signals. Speed and horsepower data were collected using the torque transducer/encoder and were recorded by hand. The bearing specification data used on the drive side and fan side are shown in Table 2.

#### 4.1.2. University of Cincinnati IMS Bearing Dataset

This dataset [43] is the life cycle data of bearings and there is a vertical and horizontal accelerometer on the housing of each bearing. There are three datasets, each containing the vibration data of four bearings. The bearing specifications used in this paper are shown in Table 3. The data information is shown in Table 4.

#### 4.1.3. v43hmbwxpm Dataset

The data come from Taihua University and the experiments were performed on the SpectraQuest Mechanical Failure Simulator (MFS-PK5M) and the data consisted of vibration signals collected from bearings with different health conditions under time-varying rotational speed conditions [50]. Data were acquired by an NI data acquisition board (NI USB-6212 BNC) for a total of 36 datasets. For each dataset, there were two experimental setups: bearing health condition and variable speed condition. The bearing health conditions included (i) healthy, (ii) inner race damage, (iii) outer race damage, (iv) rolling element damage and (v) a combination of inner race damage, outer race damage and rolling element damage. The operating speed conditions were (i) increasing speed, (ii) decreasing speed, (iii) increasing then decreasing speed and (iv) decreasing then increasing speed. Thus, there were 20 different cases for the setup. The bearing parameters are shown in Table 5. Some of the bearing failure information is shown in Table 6.

### 4.2. Dataset Selection and Division

**Figure 7:**
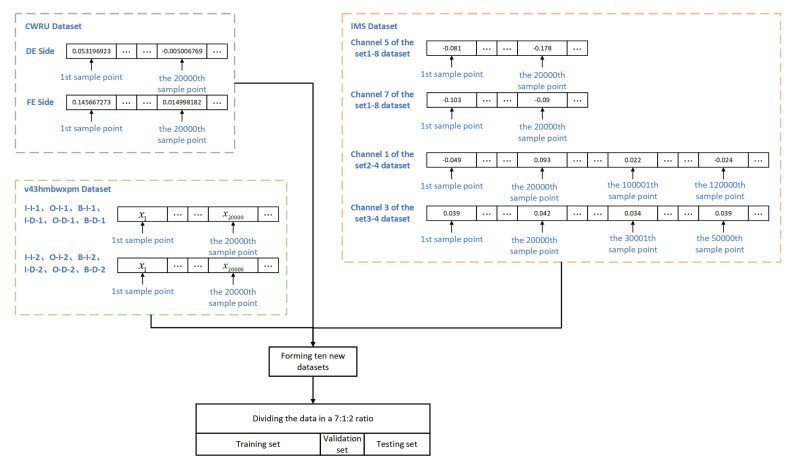
Datasets selection and division.

Select 20,000 sample points from the DE side and FE side of the CWRU dataset, respectively, to form a new dataset, the CWRU_DF dataset. In IMS data, 20,000 sample points were selected respectively from channels 5 and 7 of the datasets, sets 1–8, to form the new dataset set 1; select the 1st to 20,000th sample points and 100,001st to 200,000th sample points from channel 1 of the sets 2–4 to form the new dataset set 2; select the 1st to 20,000th sample points and 30,001st to 50,000th sample points from channel 3 of the sets 3 and 4 dataset to form a new dataset set 3. In the v43hmbwxpm data, 20,000 sample points were selected, respectively, from I-I-1 and I-I-2 of the I-I dataset to form a new dataset; other new datasets were formed in the same way. The selection of the datasets, as shown in Figure 7. The above ten datasets were divided into the training set, the validation set and the testing set in the ratio of 7:1:2, respectively.

### 4.3. Experiment and Analysis

Because the epoch times of the five models used in the experiments of this paper varies widely, other convergence properties such as the speed of loss convergence of the five models trained under the dataset are not compared.

Each network model in this paper is implemented based on Python 3.9. The operating system is a 64-bit Windows operating system with 16.00 GB of RAM and a 12th Gen Intel(R) Core(TM) i7-12700KF 3.60 GHz processor.

#### 4.3.1. Time Series Forecasting of Motor Bearing Vibration Based on Case Western Reserve University Bearing Dataset

CWRU data were selected to test the time series forecasting effects of CNN, Deep RNNs, LSTM, Transformer and Informer on data on the DE side and FE side. The data from different ends were tested to enhance the experimental results to be more accurate and convincing.

**Figure 8:**
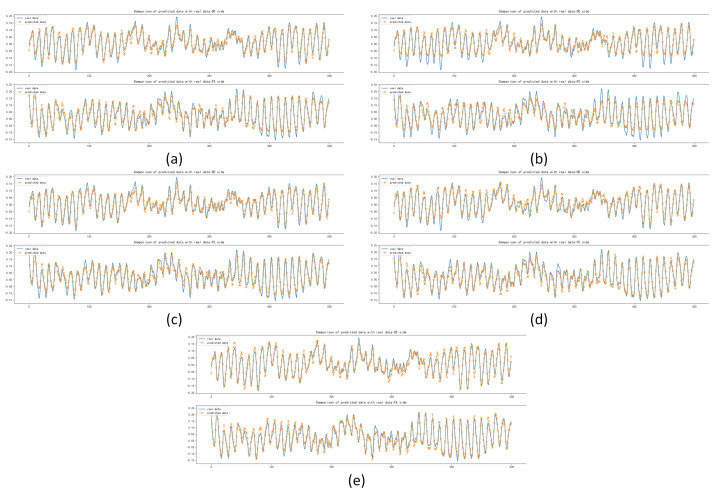
Comparison of forecasting data with real data: (**a**) Forecasting results based on CNN; (**b**) Forecasting results based on Deep RNNs; (**c**) Forecasting results based on LSTM; (**d**) Forecasting results based on Transformer; (**e**) Forecasting results based on Informer.

After the training and forecasting of the above five models, the MAE, MSE and RMSE of the above models were calculated. It was concluded that the Informer model has the best forecasting performance compared with other models, with MAE lower by $1.711\times 10^{-3}$, $6.692\times 10^{-3}$, $6.343\times 10^{-3}$ and $3.361\times 10^{-3}$, respectively; with MSE lower by $1.147\times 10^{-4}$, $5.069\times 10^{-4}$, $3.887\times 10^{-4}$ and $2.084\times 10^{-4}$, respectively; with RMSE lower by $2.511\times 10^{-3}$, $9.605\times 10^{-3}$, $7.649\times 10^{-3}$ and $4.383\times 10^{-3}$, respectively, which is shown in Table 7. The forecasting diagrams are shown in Figure 8. It can be seen from the forecasting diagrams that the five models can forecast the next 500 sample points well on the DE and FE sides, but CNN and Deep RNNs were worse and LSTM was better in forecasting extreme values. The Informer not only fitted the trend of the data correctly, but also forecast the extreme values correctly to the maximum extent, with less offset than other models and fitted the original data best among five models.

#### 4.3.2. Time Series Forecasting of Motor Bearing Vibration Based on University of Cincinnati IMS Bearing Dataset

The IMS data were selected to test the time series forecasting effect of the five models when different structures fail. Further comprehensive experiments were conducted by testing the data at the outer race of the bearing, the inner race of the bearing and the rolling element of the bearing to illustrate the forecasting ability of each model at different structures. The forecasting results of the five models used in this paper are worse under the IMS-based dataset compared to the CWRU-based dataset. The reason was that the IMS dataset has a large oscillation in the process of collecting data, which makes the collected data fluctuate more in amplitude and frequency. This problem will be the next research goal.

**Figure 9:**
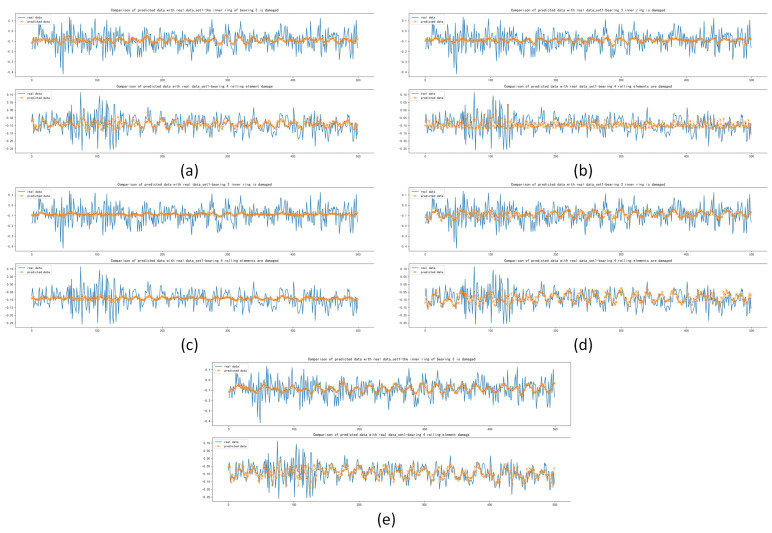
Comparison of forecasting data with real data with the damaged inner race of bearing 3 and the damaged rolling element of bearing 4: (**a**) Forecasting results based on CNN; (**b**) Forecasting results based on Deep RNNs; (**c**) Forecasting results based on LSTM; (**d**) Forecasting results based on Transformer; (**e**) Forecasting results based on Informer.

After training and forecasting of CNN, Deep RNNs, LSTM, Transformer and Informer, the MAE, MSE and RMSE of the above models were calculated. Compared with other models, the Informer had the best prediction performance, with MAE lower by $1.280\times 10^{-4}$, $1.896\times 10^{-3}$, $4.38\times 10^{-3}$ and $1.245\times 10^{-3}$ for set 1, respectively; with MSE lower by $9.900\times 10^{-6}$, $3.243\times 10^{-4}$, $7.720\times 10^{-4}$ and $2.032\times 10^{-4}$, respectively; with RMSE lower by $7.200\times 10^{-5}$, $2.306\times 10^{-3}$, $5.372\times 10^{-3}$ and $1.454\times 10^{-3}$, respectively, as shown in Table 8. The forecasting diagrams are shown in Figure 9. CNN and LSTM had the worst forecasting results with the damaged inner race of bearing 3 and the damaged rolling element of bearing 4 and they could not forecast the trend and extreme values well. It was able to forecast most of the extreme values with the damaged rolling element of bearing 4.

**Figure 10:**
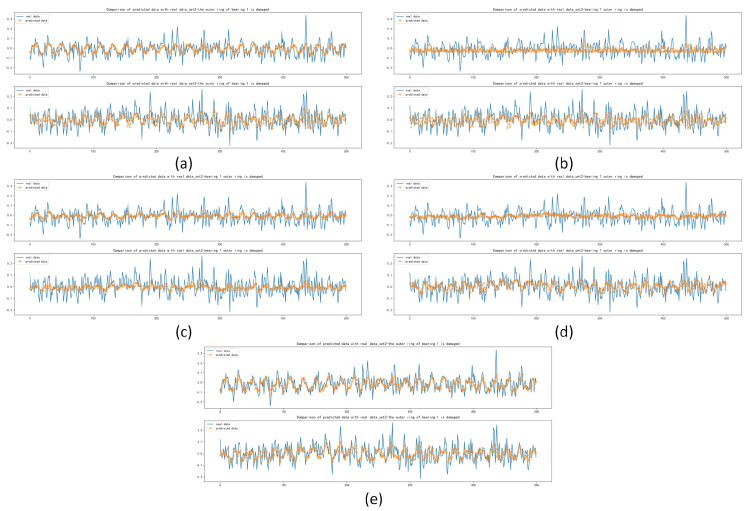
Comparison of forecasting data with real data with the damaged outer race of bearing 1: (**a**) Forecasting results based on CNN; (**b**) Forecasting results based on Deep RNNs; (**c**) Forecasting results based on LSTM; (**d**) Forecasting results based on Transformer; (**e**) Forecasting results based on Informer.

**Figure 11:**
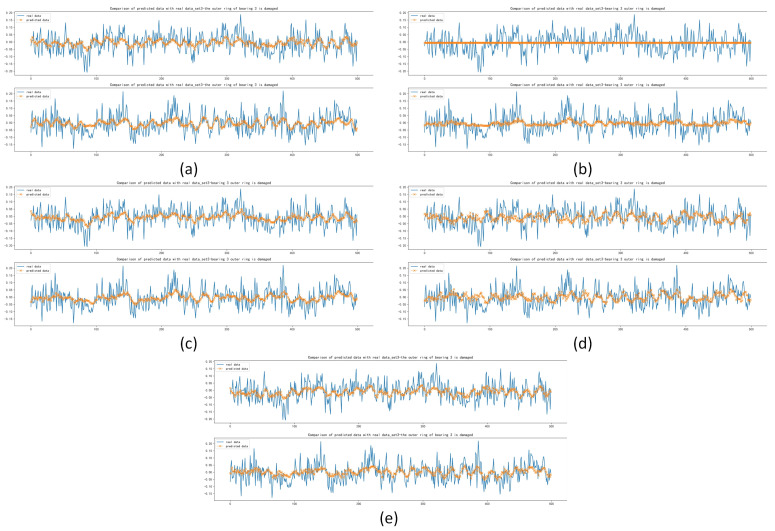
Comparison between forecasting data with real data with the damaged outer race of bearing 3: (**a**) Forecasting results based on CNN; (**b**) Forecasting results based on Deep RNNs; (**c**) Forecasting results based on LSTM; (**d**) Forecasting results based on Transformer; (**e**) Forecasting results based on Informer.

The MAE, MSE and RMSE of the Informer were slightly worse than those of CNN for set 2, with a difference of $2.710\times 10^{-4}$ for MAE, $4.050\times 10^{-4}$ for MSE and $3.25\times 10^{-4}$ for RMSE. The MAE was $4.847\times 10^{-3}$, $4.973\times 10^{-3}$ and $3.272\times 10^{-3}$ lower than the other models, respectively. The RMSE was $5.745\times 10^{-3}$, $6.068\times 10^{-3}$ and $4.133\times 10^{-3}$ lower than the other models. The calculation results of MAE, MSE and RMSE for set 3 were the best in terms of forecasting performance compared with other models. The results are shown in Table 8. By comparing the forecasting results of the five models in Figure 10 and Figure 11, it can be seen that Deep RNNs, LSTM and Transformer do not have good forecasting results in the case of damaged outer race of bearing 1 and outer race of bearing 3. The results of the Informer comparing MAE, MSE and RMSE under set 2 were not as good as those of CNN. However, it can be seen from Figure 10 that CNN did not forecast the trend and extreme values well in the first testing set of set 2, although it was improved in the second testing set, but based on these two testing sets, Informer performed better, not only forecasting the trend of the data series better but also forecasting some of the extreme values. It can be seen from Figure 10 and Figure 11 that the five models can forecast the basic trend of the data series, but the forecasting of the extreme values is poor.

#### 4.3.3. Time Series Forecasting of Motor Bearing Vibration Based on v43hmbwxpm Dataset

In this paper, the v43hmbwxpm data are selected in order to investigate the time series forecasting capability of the five models under six different conditions. These data contain data collected from the inner race, outer race and rolling element of the bearing in the accelerated condition and data collected from the inner race, outer race and rolling element of the bearing in the decelerated condition. These data were selected to complement the time series forecasting based on multiple conditions for different structures. The robustness of each model was further compared by training and testing the data to provide a strong experimental illustration for the findings of this paper.

**Figure 12:**
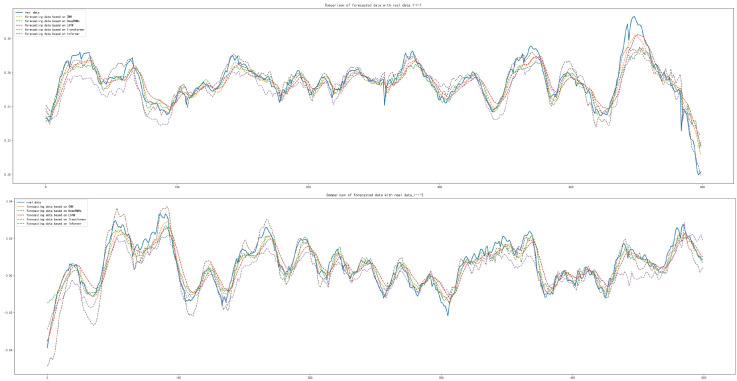
Comparison of forecasting data with real data with the damaged inner race under accelerated conditions.

**Figure 13:**
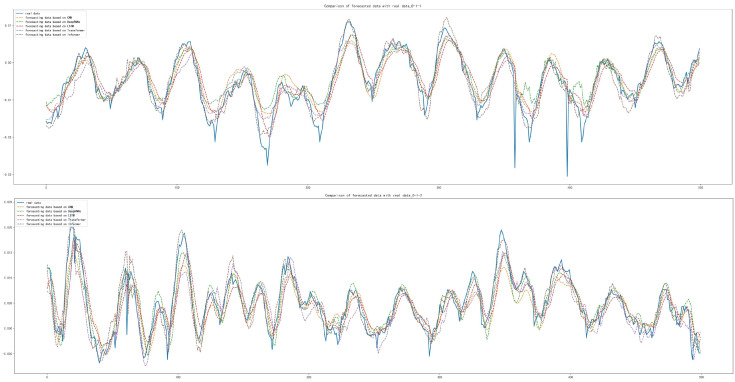
Comparison of forecasting data with real data with the damaged outer race under accelerated conditions.

**Figure 14:**
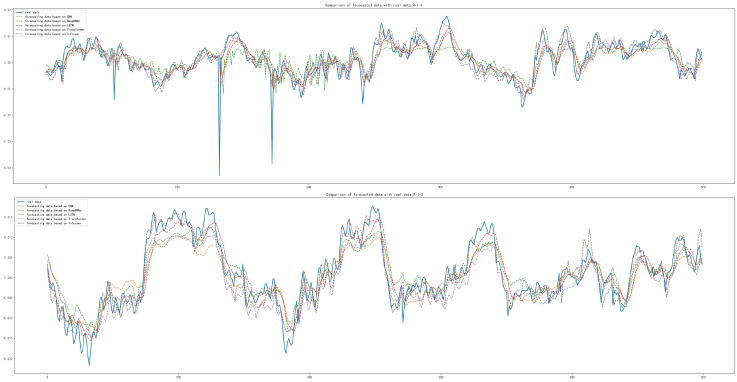
Comparison of forecasting data with real data with the damaged rolling element under accelerated conditions.

After the training and forecasting of CNN, Deep RNNs, LSTM, Transformer and Informer, the MAE, MSE and RMSE of the above models were calculated. For datasets of inner race damage (I-I), outer race damage (O-I) and rolling element damage (B-I) under accelerated conditions, compared with other models, the Informer achieved the best forecasting results, as shown in Table 9. The forecasting diagrams are shown in Figure 12 and Figure 13. The forecasting diagrams show that Transformer has poor forecasting results, while CNN, Deep RNNs and LSTM are able to forecast the data transformation trends and some of the extreme values, but their forecasting results had a certain offset. Compared with the other models, Informer had the best forecasting results, which can not only forecast the trend of data series transformation and extreme values better, but also has less offset. The forecasting diagrams of the dataset (B-I) with damaged rolling element forecast under the accelerated condition are shown in Figure 14. CNN, Deep RNNs and LSTM are able to forecast the trend of data series, but they are not better than Transformer, which is not specifically designed for the time series forecasting. Informer was closest to the real data in terms of trend and also forecast most of the extreme values with minimal offset.

**Figure 15:**
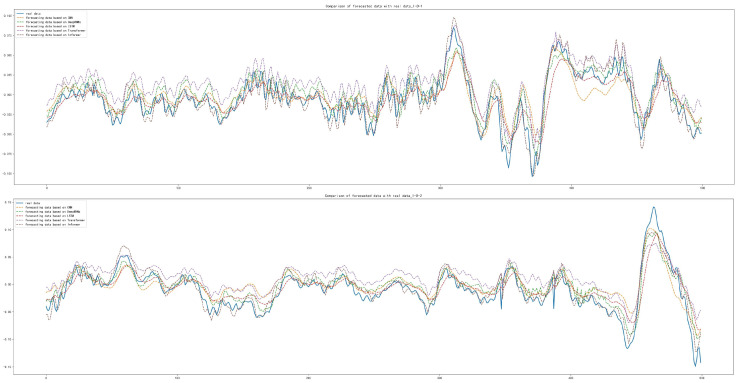
Comparison of forecasting data with real data with the damaged inner race under decelerated conditions.

**Figure 16:**
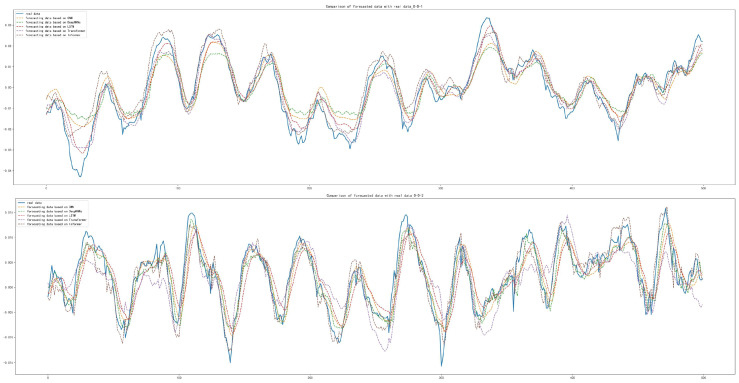
Comparison of forecasting data with real data with the damaged outer race under decelerated conditions.

The prediction results for the inner race damage dataset (I-D) under decelerated conditions and the outer race damage (O-D) dataset under decelerated conditions showed that Informer achieved the best forecasting results compared to the other models, which is shown in Table 10. The forecasting diagrams are shown in Figure 15 and Figure 16. It can be seen from Figure 15 that the Transformer model has a better forecasting effect of the data series trend, but there is an overall upward shift. CNN, Deep RNNs and LSTM are found to have poorer forecasting results for the trend and extreme values of the data series, compared with Informer which has a better fit with the real data.

**Figure 17:**
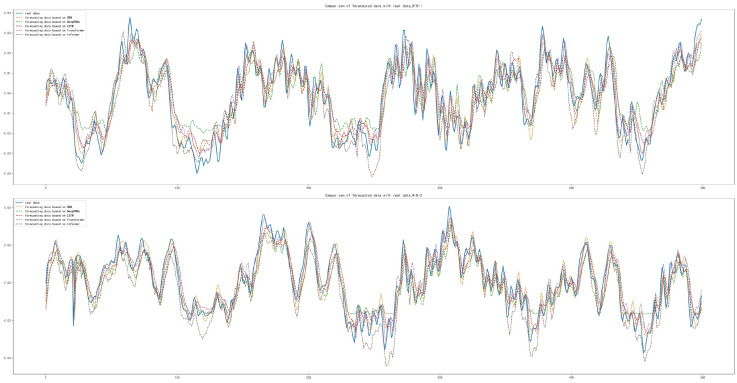
Comparison of forecasting data with real data with the damaged rolling element under decelerated conditions.

The MAE, MSE and RMSE of Informer based on the rolling element damage (B-D) dataset under decelerated conditions were slightly worse than those of CNN and Transformer; the difference of MAE is $1.243\times 10^{-3}$ and $1.261\times 10^{-3}$, respectively; the difference of MSE is $2.030\times 10^{-3}$ and $1.948\times 10^{-3}$, respectively; and the difference of RMSE is $1.623\times 10^{-3}$ and $1.548\times 10^{-3}$, respectively. Compared with Deep RNNs and LSTM, the MAE of the forecasting results are lower by $4.377\times 10^{-4}$ and $6.674\times 10^{-4}$, respectively; the MSE lower by $9.361\times 10^{-6}$ and $1.056\times 10^{-5}$, respectively; and the RMSE lower by $6.340\times 10^{-3}$ and $7.113\times 10^{-3}$, respectively, as shown in Table 10. The forecasting diagrams are shown in Figure 17, from which it can be seen that Deep RNNs and LSTM have offsets in the data sequence forecasting and some extreme values are not well forecasted. Compared with CNN and Transformer, Informer has a small difference in the forecasting of the change trend of the data series and the offset of its own forecasting results is small. The offset of individual extreme value forecasting is relatively large, so the calculation results of MAE, MSE and RMSE are not as good as these two models.

## 5. Conclusions

The motor is the core equipment of the power station and time series forecasting of motor bearing vibration is a crucial step in bearing fault diagnosis, bearing remaining service life prediction, etc. Therefore, we specialize in research on time series forecasting of motor bearing vibration. In this paper, Informer is innovatively introduced into time series forecasting of motor bearing vibration and the model structure is optimized and the parameters of Informer are optimized by applying random search. The datasets CWRU, IMS and v43hmbwxpm were used for time series forecasting of motor bearing vibration and the experimental results were analyzed. The analysis showed that, compared to the existing work, Informer is able to forecast the future time series quickly and accurately when facing inner race damage, outer race damage and rolling element damage. Superior results can still be obtained for damage under accelerated or decelerated conditions, with better forecasting results for data-series trends and extreme values of data. It had excellent performance in evaluation indexes such as MAE, MSE and RMSE and the forecasting results. The forecasting of conventional models is prone to certain offset, while the forecasting results of the method proposed in this paper were more closely matched to the real data and this method reduced the error accumulation in forecasting and improved the model forecasting performance. It can be used for sensing technology monitoring.

In the future, we will conduct study and research concerning time series forecasting methods. Deeper research on data with oscillation, fluctuation amplitude and fluctuation frequency will be carried out and the impact of this problem on the forecasting operation will be solved. Self-testing data will be added in future experiments to further improve the persuasiveness of the model. Bearing fault diagnosis or bearing remaining useful life prediction will be taken as the next directions of research.

## Figures and Tables

**Table 1:** Network model parameters.

**Batch Size**	16	**Epochs**	10
**Activation Function**	GELU	**Learning Rate**	0.0001
**Encoder Input Size**	7	**Decoder Input Size**	7
**Encoder Layer**	2	**Decoder Layer**	1
**Time Feature Encoding**	hour	**Dropout**	0.02
**Loss Function**	mse	**Forecasting Length**	500

**Table 2:** The bearing specification data used in the dataset.

Bearing Position	Bearing Type	Inner Race Diameter (mm)	Outer Race Diameter (mm)	Thickness (mm)	Rolling Element Diameter (mm)	Pitch Diameter (mm)	Sampling Frequency
Drive Side	6205-2RS JEM SKF Deep Groove Ball Bearings	25	52	15	7.94	39.04	12 kHz 48 kHz
Fan Side	6203-2RS JEM SKF Deep Groove Ball Bearings	17	40	12	6.7462	28.4988	12 kHz

**Table 3:** The bearing specification data used in dataset.

Bearing Type	Static Load (lbs)	Contact Angle	Number of Scrolling Bodies per Row	Rolling Element Diameter (mm)	Pitch Diameter (mm)
Rexnord ZA-2115	6000	15.17${}^{\circ }$	16	8.4	71.5

**Table 4:** Dataset Description.

Number of Signal Channels	Bearing 1	Bearing 2	Bearing 3	Bearing 4	Sampling Frequency	Abnormal
8	Channel 1 and Channel 2	Channel 3 and Channel 4	Channel 5 and Channel 6	Channel 7 and Channel 8	Once every 10 m (the first 43 files collected every 5 m)	Damaged inner race of bearing 3 and damaged rolling elements of bearing 4
4	Channel 1	Channel 2	Channel 3	Channel 4	Once every 10 m	Damaged outer race of bearing 1
4	Channel 1	Channel 2	Channel 3	Channel 4	Once every 10 m	Damaged outer race of bearing 3

**Table 5:** Bearing parameters.

Bearing Type	Pitch Diameter (mm)	Ball Diameter (mm)	Number of Balls	BPFI ($f_{r}$)	BPFO ($f_{r}$)	Sampling Frequency
ER16K	38.52	7.94	9	5.43	3.57	200 kHz

**Table 6:** Bearing damaged information.

Bearing Condition	Increasing Speed		Decreasing Speed	
**Damaged inner race**	**I-I**	I-I-1	**I-D**	I-D-1
		I-I-2		I-D-2
		I-I-3		I-D-3
**Damaged outer race**	**O-I**	O-I-1	**O-D**	O-D-1
		O-I-2		O-D-2
		O-I-3		O-D-3
**Damaged rolling element**	**R-I**	R-I-1	**R-D**	R-D-1
		R-I-2		R-D-2
		R-I-3		R-D-3

**Table 7:** Time series forecasting results for CWRU datasets.

Metric	CNN [15]	Deep RNNs [38]	LSTM [17]	Transformer [31]	Informer
MAE	$1.8874\times 10^{-2}$	$2.3855\times 10^{-2}$	$2.3506\times 10^{-2}$	$2.0524\times 10^{-2}$	$1.7163\times 10^{-2}$
MSE	$5.8046\times 10^{-4}$	$9.7260\times 10^{-4}$	$8.5439\times 10^{-4}$	$6.7416\times 10^{-4}$	$4.6574\times 10^{-4}$
RMSE	$2.4092\times 10^{-2}$	$3.1186\times 10^{-2}$	$2.9230\times 10^{-2}$	$2.5964\times 10^{-2}$	$2.1581\times 10^{-2}$
Running Time (s)	480	600	500	5726	162

**Table 8:** Time series forecasting results for IMS datasets.

Dataset	Metric	CNN [15]	Deep RNNs [38]	LSTM [17]	Transformer [31]	Informer
**Set1**	MAE	$5.2431\times 10^{-2}$	$5.4199\times 10^{-2}$	$5.6683\times 10^{-2}$	$5.3548\times 10^{-2}$	$5.2303\times 10^{-2}$
	MSE	$4.7943\times 10^{-3}$	$5.1087\times 10^{-3}$	$5.5564\times 10^{-3}$	$4.9876\times 10^{-3}$	$4.7844\times 10^{-3}$
	RMSE	$6.9241\times 10^{-2}$	$7.1475\times 10^{-2}$	$7.4541\times 10^{-2}$	$7.0623\times 10^{-2}$	$6.9169\times 10^{-2}$
	Running Time (s)	480	600	400	4451	176
**Set2**	MAE	$4.8317\times 10^{-2}$	$4.9114\times 10^{-2}$	$5.3561\times 10^{-2}$	$5.1860\times 10^{-2}$	$4.8588\times 10^{-2}$
	MSE	$3.8702\times 10^{-3}$	$3.9699\times 10^{-3}$	$4.7065\times 10^{-3}$	$4.4446\times 10^{-3}$	$3.9107\times 10^{-3}$
	RMSE	$6.2211\times 10^{-2}$	$6.3007\times 10^{-2}$	$6.8604\times 10^{-2}$	$6.6669\times 10^{-2}$	$6.2536\times 10^{-2}$
	Running Time (s)	480	750	800	5999	177
**Set3**	MAE	$4.8944\times 10^{-2}$	$5.2173\times 10^{-2}$	$5.0191\times 10^{-2}$	$5.0256\times 10^{-2}$	$4.7281\times 10^{-2}$
	MSE	$3.8858\times 10^{-3}$	$4.4246\times 10^{-3}$	$4.0997\times 10^{-3}$	$4.0718\times 10^{-3}$	$3.9268\times 10^{-3}$
	RMSE	$6.2336\times 10^{-2}$	$6.6518\times 10^{-2}$	$6.4029\times 10^{-2}$	$6.3811\times 10^{-2}$	$6.2664\times 10^{-2}$
	Running Time (s)	480	825	800	5578	164

**Table 9:** Time series forecasting results under accelerated conditions.

Dataset	Metric	CNN [15]	Deep RNNs [38]	LSTM [17]	Transformer [31]	Informer
**I-I**	MAE	$4.9994\times 10^{-3}$	$4.9735\times 10^{-3}$	$5.2031\times 10^{-3}$	$6.1465\times 10^{-3}$	$4.9413\times 10^{-3}$
	MSE	$5.2178\times 10^{-5}$	$5.9317\times 10^{-5}$	$5.1365\times 10^{-5}$	$6.9594\times 10^{-5}$	$4.5778\times 10^{-5}$
	RMSE	$7.2234\times 10^{-3}$	$7.7017\times 10^{-3}$	$7.1670\times 10^{-3}$	$8.3423\times 10^{-3}$	$6.7659\times 10^{-3}$
	Running Time (s)	480	2475	400	4252	155
**O-I**	MAE	$2.2311\times 10^{-3}$	$2.1050\times 10^{-3}$	$2.3986\times 10^{-3}$	$2.1532\times 10^{-3}$	$1.7147\times 10^{-3}$
	MSE	$9.5604\times 10^{-6}$	$9.5604\times 10^{-6}$	$1.0922\times 10^{-5}$	$8.9571\times 10^{-6}$	$6.2589\times 10^{-6}$
	RMSE	$3.0920\times 10^{-3}$	$3.0825\times 10^{-3}$	$3.3048\times 10^{-3}$	$2.9928\times 10^{-3}$	$2.5018\times 10^{-3}$
	Running Time (s)	480	2625	400	4344	166
**R-I**	MAE	$2.9234\times 10^{-3}$	$3.2357\times 10^{-3}$	$2.8091\times 10^{-3}$	$2.6975\times 10^{-3}$	$2.1812\times 10^{-3}$
	MSE	$1.5228\times 10^{-5}$	$1.8761\times 10^{-5}$	$1.3894\times 10^{-5}$	$1.3384\times 10^{-5}$	$9.5485\times 10^{-6}$
	RMSE	$3.9023\times 10^{-3}$	$4.3314\times 10^{-3}$	$3.7274\times 10^{-3}$	$3.6584\times 10^{-3}$	$3.0901\times 10^{-3}$
	Running Time (s)	480	2775	400	4293	158

**Table 10:** Time series forecasting results under decelerated conditions.

Dataset	Metric	CNN [15]	Deep RNNs [38]	LSTM [17]	Transformer [31]	Informer
**I-D**	MAE	$2.1981\times 10^{-2}$	$1.8724\times 10^{-2}$	$2.1514\times 10^{-2}$	$2.2651\times 10^{-2}$	$1.4192\times 10^{-2}$
	MSE	$1.1893\times 10^{-3}$	$9.2163\times 10^{-4}$	$1.3191\times 10^{-3}$	$1.0206\times 10^{-3}$	$5.2582\times 10^{-4}$
	RMSE	$3.4487\times 10^{-2}$	$3.0358\times 10^{-2}$	$3.6320\times 10^{-2}$	$3.1947\times 10^{-2}$	$2.2931\times 10^{-2}$
	Running Time (s)	480	2700	400	4295	169
**O-D**	MAE	$3.6730\times 10^{-3}$	$3.9415\times 10^{-3}$	$3.6925\times 10^{-3}$	$3.9617\times 10^{-3}$	$2.8098\times 10^{-3}$
	MSE	$2.7954\times 10^{-5}$	$3.5443\times 10^{-5}$	$2.5712\times 10^{-5}$	$3.3534\times 10^{-5}$	$2.0841\times 10^{-5}$
	RMSE	$5.2872\times 10^{-3}$	$5.9534\times 10^{-3}$	$5.0708\times 10^{-3}$	$5.7908\times 10^{-3}$	$4.5652\times 10^{-3}$
	Running Time (s)	480	2550	400	4344	171
**R-D**	MAE	$4.2048\times 10^{-3}$	$5.8855\times 10^{-3}$	$6.1152\times 10^{-3}$	$4.1870\times 10^{-3}$	$5.4478\times 10^{-3}$
	MSE	$2.9625\times 10^{-5}$	$5.9285\times 10^{-5}$	$6.0482\times 10^{-5}$	$3.0447\times 10^{-5}$	$4.9924\times 10^{-5}$
	RMSE	$5.4429\times 10^{-3}$	$7.6997\times 10^{-3}$	$7.7770\times 10^{-3}$	$5.5179\times 10^{-3}$	$7.0657\times 10^{-3}$
	Running Time (s)	480	2475	400	4451	182

## Data Availability

‘The Case Western Reserve University Bearing Dataset’ at https://engineering.case.edu/bearingdatacenter/welcome (accessed on 15 May 2022). ‘The University of Cincinnati IMS Bearing Dataset’ at http://ti.arc.nasa.gov/projects/data_prognostics (accessed on 15 May 2022). ‘The v43hmbwxpm Dataset’ at https://data.mendeley.com/datasets/v43hmbwxpm/1 (accessed on 15 May 2022).
